# Correlates of unprotected anal sex among men who have sex with men in Tijuana, Mexico

**DOI:** 10.1186/1471-2458-12-433

**Published:** 2012-06-13

**Authors:** Sergio Barrón-Limón, Shirley J Semple, Steffanie A Strathdee, Remedios Lozada, Adriana Vargas-Ojeda, Thomas L Patterson

**Affiliations:** 1 National Center for the Prevention of HIV & AIDS (CENSIDA), Mexico City, Mexico; 2 Department of Psychiatry, University of California, San Diego, La Jolla, CA, USA; 3 Division of Global Public Health, Department of Medicine, University of California, San Diego, La Jolla, CA, USA; 4 Ministry of Health, State of Baja California, Tijuana, Mexico; 5 Autonomous University of Baja California, Tijuana, Mexico

**Keywords:** Men who have sex with men, HIV risk behaviors, Unprotected anal intercourse, Mexico

## Abstract

**Background:**

Although men who have sex with men (MSM) are disproportionately affected by HIV/AIDS in Mexico, data on current risk behaviors in this population are lacking. This study investigated the prevalence and correlates of unprotected anal intercourse (UAI) in a sample of 260 MSM in Tijuana, Mexico.

**Methods:**

In June 2010, men attending a gay pride celebration were invited to complete a sexual risk survey. Men who reported UAI with a male partner in the past year were compared with men who reported only protected anal sex during the same period.

**Results:**

Mean age of participants was 29.7; 54% had a high school diploma or less; and 43% were unemployed. In the past year, 55% had been tested for HIV, 21% reported using illicit drugs before or during sex, and 94% had sex only with men. Overall, 50% reported having UAI with another male in the past year. Factors independently associated with UAI in the past year were unemployment (AOR = 1.87), attending adult movie theaters (AOR = 2.21), using illicit drugs before or during sex (AOR = 2.43), and not having a recent HIV test (AOR = 1.85).

**Conclusions:**

Interventions to promote HIV testing and condom use among men who have sex with men may want to consider venue-specific approaches, as well as focus on drug-use issues in the context of unsafe sex.

## Background

In Mexico, the largest percentage of persons living with AIDS are men who have sex with men (MSM). In 2007, unprotected sex between men accounted for 40.3% of all new sexually transmitted AIDS cases in Mexico [1]. HIV prevalence among MSM was estimated to be between 10% and 13.5% in 2006 [2].

Baja California has the second highest cumulative AIDS incidence among Mexico’s 32 states. Tijuana is Baja California’s largest city, with approximately 1.4 million inhabitants. It is located adjacent to San Diego, California, on the U.S. border, and it is experiencing an escalating HIV epidemic. Recent data on HIV seroprevalence among MSM in Tijuana are lacking. Ruiz reported an HIV seroprevalence rate of 19% in a sample of 249 MSM aged 18–29 living in Tijuana. Less than 50% of the Tijuana sample had ever been tested for HIV, and 43% had never received any HIV prevention education. A comparison of the Tijuana sample with San Diego-residing MSM revealed that Tijuana MSM were significantly more likely to report sex with females and injection drug use [3]. Despite a national HIV strategy and substantial efforts to stop the spread of HIV/AIDS in Mexico, data are scarce to inform HIV prevention and intervention programs for MSM in Tijuana.

Research with MSM in the U.S. and other developed countries has identified a number of risk factors for UAI, including socio-demographic characteristics such as age and ethnicity [4], sexual partner type [5,6], venues where MSM go to meet sex partners [7], and illicit drug use before and during sex [8-10]. Since little is known about factors associated with UAI among MSM in Mexico, we examined the prevalence and correlates of unprotected anal sex (UAI) in a sample of MSM who frequented gay establishments in Tijuana. The findings from this study will help to inform and direct HIV prevention efforts for MSM in the Tijuana border region.

## Methods

### Sample recruitment and data collection

MSM in Tijuana, Baja California who attended a gay pride event on June 19–20, 2010 called “Inclusion in Revolution,” which was held at the Plaza Santa Cecilia in downtown Tijuana, were approached by volunteers to participate in an anonymous survey. The survey was administered during the festival. Social networks and electronic and written media were used to inform men of when and where the survey would be administered. Two male outreach workers screened potential participants for eligibility. Eligibility criteria were: at least 18 years of age, self-identified as a man who had anal or oral sex with another man in the past year, a resident of Tijuana or frequent visitor to gay establishments in Tijuana, and able to read and understand Spanish. Signed consent to study participation was obtained from all eligible participants. The survey was self-administered, and participants were provided a private space at the event to complete the survey. To ensure confidentiality, respondents were given an envelope into which they could seal the completed survey. As a thank-you gift, respondents were given a package of condoms provided by the AIDS Health Care Foundation and one of the following incentives: key ring, pen, shirt, mug, or sticker. The research described in this paper was carried out in compliance with the Helsinki Declaration, and the protocol was reviewed and approved by the Human Research Protections Program at the University of California, San Diego (Project #110451).

### Study measures

The questionnaire was developed by the first author in a methodology class for the Masters of Public Health Program at Universidad Autónoma de Baja California (UABC). It was adapted from men’s health questionnaires developed by Grupo Compañeros Ciudad Juárez and Intersex 2006 (EMIS) in Spain. The questionnaire covered a range of topics including socio-demographic factors, venues attended to meet sex partners, illicit drugs used before and during sex, sexual practices with men and women, condom use, sexual partner types, HIV serostatus, and HIV testing. The recall period for most behaviors was the last year. Questions regarding anal sex with men were asked separately for receptive and insertive sex (e.g., In the past year, how often did another man penetrate you during anal sex?). A follow-up question asked how often a condom was used for these two types of anal sex (e.g., When other men penetrated you anally, how often did the man who penetrated you wear a condom?). A summary variable was calculated to represent total number of unprotected anal sex acts in the past year. Questions regarding partner types were worded “In the past year, in your relationship with men, did you have sex with a casual partner (pareja ocasional)? With a steady partner (pareja estable)?” The question regarding sexually transmitted infections (STIs) was worded as follows: “Have you been diagnosed with a sexually transmitted infection during the past year?”

### Statistical analyses

Men who reported UAI with a male partner in the past year were compared with their counterparts who reported only protected anal sex with a male partner during the past year. Continuous variables were examined using student’s *t*-test, and categorical variables were examined using the chi-square test. Univariate and multivariate logistic regression analyses were performed to identify factors associated with UAI. Factors that attained a significance level of < 5% in univariate analyses were included in the multivariate model. A significance level of *p* < .05 was also used in the multivariate analysis.

## Results

The questionnaire was completed by a convenience sample of 305 Spanish-speaking men who attended the “Inclusion for Revolution” festival in Tijuana, Mexico in June 2010. The festival was Tijuana’s first LGBT (lesbian, gay, bisexual, transgender) community event, which included dancing, food, and seminars on gay and transgender rights [11]. The event was modeled after San Diego’s annual Pride Festival and Parade and was attended by persons from the LGBT community and the general public from both Mexico and the U.S. Data from 45 participants were excluded from the present analyses for two reasons: questions regarding anal sex were not answered (*N* = 39) or participants reported no anal sex with a man in the past year (*N* = 6). Our analytic sample thus consisted of 260 men who reported having anal sex with another man in the past year.

Eligible and ineligible participants did not differ significantly in age, education, income, employment status, or number of years residing in Tijuana; however, participants who were excluded from the analysis were significantly more likely to self-identify as heterosexual (9.1% vs. 1.5%, χ^2^ = 21.0, *P* < .001) and to report having had only female partners in the past year (15.6% vs. 0.8%, χ^2^ = 30.8, *P* < .001).

Eighty-eight percent of the analytic sample self-identified as homosexual, and 94% reported having had sex only with men in the past year. Seventy-five percent had lived in Tijuana for five or more years. Fifty-four percent had a high school diploma or less, and the average income was 9 468 pesos ($697 US) per month. Participants ranged in age from 18 to 65 years (mean = 29.7; standard deviation *[SD]* = 8.0). Forty-three percent were unemployed. Approximately 7% reported their serostatus as HIV-positive; 62% and 31% reported their serostatus as HIV-negative or unknown, respectively. Approximately 7% reported being paid for sex by a male partner in the past year.

Fifty percent reported UAI with a male partner in the past year, and 50% reported only protected anal sex with male partners. Men who had unprotected anal sex were significantly more likely to be unemployed compared to their counterparts who had only protected anal sex (see Table 1).

**Table 1:** Background characteristics of 260 Tijuana-residing men who reported having anal sex with a man

**Variable**	**Total sample (*N* = 260)**	**Had unprotected anal sex in past year (*N* = 129)**	**No unprotected anal sex in past year (*N* = 131)**
Age in years (mean, *SD*)	29.7 (8.0)	30.1 (8.4)	29.2 (7.5)
Income per month in pesos (mean, *SD*)	9 468 (10 512)	9 598 (11 389)	9 340 (9 670)
Number of years resident of Tijuana			
Less than 1 year	18 (7.1%)	9 (7.2%)	9 (7.0%)
1 to 5 years	46 (18.1%)	24 (19.2%)	22 (17.0%)
5 to 9 years	30 (11.8%)	15 (12.0%)	15 (11.6%)
10 years or more	160 (63.0%)	77 (61.6%)	83 (64.4%)
Educational attainment			
Elementary school	3 (1.2%)	3 (2.3%)	0 (0.0%)
Junior high school	45 (17.4%)	22 (17.1%)	23 (17.7%)
Technical degree	17 (6.6%)	10 (7.8%)	7 (5.4%)
High school	75 (28.9%)	31 (24.0%)	44 (33.8%)
Bachelor’s degree	100 (38.6%)	52 (40.3%)	48 (36.9%)
Specialty, master’s or doctoral degree	19 (7.3%)	11 (8.5%)	8 (6.2%)
Percent employed^a^	146 (56.6%)	64 (50.0%)	82 (63.1%)
Gender of sexual attraction^b^			
Women only	5 (1.9%)	3 (2.3%)	2 (1.5%)
Both men and women	29 (11.2%)	17 (13.2%)	12 (9.2%)
Men only	223 (86.1%)	108 (83.7%)	115 (88.5%)
Not sure	2 (0.8%)	1 (0.8%)	1 (0.8%)
Gender of sex partner(s) in past year			
Women only	2 (0.8%)	0 (0.0%)	2 (1.5%)
Both men and women	13 (5.0%)	4 (3.1%)	9 (6.9%)
Men only	244 (94.2%)	125 (96.9%)	119 (91.6%)
Sexual orientation			
Heterosexual only	4 (1.5%)	1 (0.8%)	3 (2.3%)
Bisexual	28 (10.8%)	15 (11.6%)	13 (10.0%)
Homosexual only	227 (87.6%)	113 (87.6%)	114 (87.7%)

All data shown are in the format *No. (%)* unless otherwise noted. *SD* = standard deviation.

^a^*P* < 0.05.

^b^ Participants were asked, “To which gender are you most sexually attracted?”

### Sexual risk venues

The most frequently reported venues attended by MSM in the past year with the intention of meeting male sex partners were (in rank order): nightclubs and discos (85.8%), bars (78.6%), gay websites (73.4%), cafés (66.7%), and internet café, sex shop, or booths (40.6%). As shown in Table 2, MSM who had UAI with male partners in the past year were significantly more likely to report attendance at an adult movie theater or dark room/appointment home/hotel-motel compared to men who had only protected anal sex during this time period. The two groups did not differ in attendance at any other venues for meeting sexual partners.

**Table 2:** Venues attended in order to meet male sex partners

**Attended the following types of venue1 or more times in past year:**	**Total sample (*N* = 260)**	**Had unprotected anal sex in past year(*N* = 129)**	**No unprotected anal sex in past year(*N* = 131)**
Night club or disco	211 (85.8%)	103 (85.8%)	108 (85.7%)
Bar	191 (78.6%)	96 (80.7%)	95 (76.6%)
Café	144 (66.7%)	66 (61.7%)	78 (71.6%)
Steam bath or sauna	79 (34.6%)	41 (36.9%)	38 (32.5%)
Adult movie theater^a^	54 (23.1%)	35 (30.4%)	19 (16.0%)
Dark room, appointment home, hotel-motel^b,^	88 (37.3%)	52 (44.1%)	36 (30.5%)
Public highway	85 (37.0%)	41 (36.0%)	44 (37.9%)
Gay website	174 (73.4%)	88(75.9%)	86 (71.1%)
Internet café, sex shop, booth	93 (40.6%)	43 (39.1%)	50 (42.0%)
Restroom in a public place		45 (19.7%)	25 (22.3%)	20 (17.2%)

All data shown are in the format *No. (%)*.

^a^*P* < 0.01.

^b^*P* < 0.05.

### Sexual risk factors

MSM who reported UAI with male partners in the past year differed from those who engaged in only protected anal sex on three major domains. First, MSM who engaged in UAI were significantly more likely to have paid a male partner for sex in the past year compared to MSM who always engaged in protected anal sex. Second, MSM reporting UAI were also significantly more likely to have used alcohol or illicit drugs before or during sex with a male partner in the past year compared to men who always used condoms for anal sex. Third, a higher percentage of men in the UAI group reported not having been tested for HIV in the past year compared to their counterparts who always used condoms for anal sex (see Table 3).

**Table 3:** Sexual and drug risk behaviors of men who reported anal sex with a male

	**Total sample (*N* = 260)**	**Had unprotected anal sex in past year(*N* = 129)**	**No unprotected anal sex in past year(*N* = 131)**
Age when first had sex with a man (mean, SD)	16.5 (4.3)	16.4 (4.0)	16.7 (4.7)
Number of men had sex with in past year (mean, SD)	5.1 (8.1)	6.0 (9.8)	4.3 (6.0)
Paid a male partner for sex in past year	24 (9.3%)	14 (10.9%)	10 (7.7%)
Was paid by a male partner for sex in past year^a^	18 (6.9%)	13 (10.1%)	5 (3.8%)
Had a steady male partner in past year	163 (63.7%)	75 (59.5%)	88 (67.7%)
Had a casual male partner in past year	97 (38.0%)	53 (42.1%)	44 (34.1%)
Used alcohol before or during sex with a male in past year^a^	196 (75.7%)	104 (80.6%)	92 (70.8%)
Used any illicit drug before or during sex with a male in past year^b^	55 (21.3%)	38 (29.7%)	17 (13.1%)
Used marijuana	43 (16.7%)	22 (17.1%)	21 (16.4%)
Used poppers	21 (8.1%)	14 (10.9%)	7 (5.4%)
Used amphetamines	2 (0.8%)	2 (1.6%)	0 (0.0%)
Used cocaine	12 (4.7%)	8 (6.2%)	4 (3.1%)
Used heroin	3 (1.2%)	2 (1.6%)	1 (0.8%)
Used Ecstasy^a^	10 (3.9%)	8 (6.2%)	2 (1.6%)
Diagnosed with STI in past year	31 (12.2%)	20 (15.7%)	11 (8.7%)
Tested for HIV in past year^a^	139 (54.7%)	61 (48.0%)	78 (61.4%)

All data shown are in the format *No. (%)* unless otherwise noted. SD = standard deviation.

^a^*P* < .05.

^b^*P* < .001.

### Illicit drug use

Marijuana and poppers (amyl nitrates) were the two most common illicit drugs used before or during sex with male partners (16.7% and 8.1%, respectively). Cocaine and ecstasy use were reported by 4.7% and 3.9% of the sample, respectively. The UAI and non-UAI groups differed on only one drug use variable: MSM who reported using ecstasy before or during sex with a male partner were significantly more likely to have had UAI in the past year compared to men who reported only protected anal sex (see Table 3).

### Factors associated with unprotected anal sex

MSM who were unemployed had about twice the odds of having had unprotected anal sex in the past year (odds ratio *[OR]* = 1.71, 95% confidence interval *[CI]* = 1.04–2.81). MSM who reported going to adult movie theaters to meet sex partners had approximately twice the odds of having had unprotected anal sex with a man in the past year (*OR* = 2.30, 95% *CI* 1.23–4.33). MSM who attended dark room/appointment home/hotel-motel to find sex partners also had a greater odds of having unprotected anal sex with male partners (*OR* = 1.80, 95% *CI* 1.05–3.06). Moreover, MSM who reported using illicit drugs before or during sex with male partners had about three times the odds of having had UAI with men in the past year (*OR* = 2.81, 95% *CI* 1.49–5.30). Finally, MSM who had not been tested for HIV in the past year more likely to have had unprotected anal sex (*OR* = 1.72, 95% *CI* 1.05–2.84) (Table 4).

**Table 4:** Factors associated with unprotected anal sex among 260 men who have sex with men

	**Odds ratio (95% CI)**	**Adjusted odds ratio**^**a**^**(95% CI)**
Background characteristics		
Age (per year increase)	1.02 (0.98–1.05)	
Number of years resident of Tijuana(< 1 year versus ≥ 1 year)	0.97 (0.37–2.52)	
Education (High school or less versus some college or more)	0.89 (0.48–1.67)	
Monthly income in pesos (< 20000 versus ≥ 20000 pesos)	0.58 (0.27–1.26)	
Unemployed (versus employed)	1.71^b^ (1.04–2.81)	1.87^b^ (1.06–3.31)
Sexual orientation (gay versus other)	0.99 (0.47–2.08)	
Gender of sex partners in past year (men only versus other)	2.76 (0.95–7.97)	
Gender of sexual attraction (men only versus other)	0.67 (0.33–1.37)	
Venues attended to meet male sexual partners in past year		
Night club or disco	1.01 (0.49–2.07)	
Bar	1.27 (0.69–2.36)	
Café	0.64 (0.36–1.13)	
Steam bath or sauna	1.22 (0.71–2.10)	
Adult movie theater	2.30^c^ (1.23–4.33)	2.21^b^ (1.09–4.51)
Dark room^d^, appointment home^e^,hotel, motel	1.80^b^ (1.05–3.06)	
Public highway	0.92 (0.54 - 1.57)	
Gay website	1.28 (0.72–2.28)	
Internet café, sex shop, booth	0.89 (0.52–1.50)	
Restroom in public location	1.38 (0.72–2.66)	
Illicit drugs used before or during sex with male partners in past year		
Marijuana	1.05 (0.54–2.02)	
Poppers	2.12 (0.83–5.44)	
Amphetamines	3.05 (0.31–29.7)	
Cocaine	2.07 (0.61–7.04)	
Heroin	2.02 (0.18–22.5)	
Ecstasy	4.20 (0.87–20.2)	
Sexual risk behaviors		
Age when first had sex with a man (< 21 years versus ≥ 21 years)	0.86 (0.43–1.73)	
Number of male sex partners in past year (< 7 versus ≥ 7 male partners)	1.67 (0.82–3.42)	
Paid a male partner for sex in past year	1.46 (0.62–3.42)	
Paid by a male partner for sex in the past year	2.80 (0.97–8.10)	
Had steady male partner in past year	0.70 (0.42–1.17)	
Had casual male partner in past year	1.40 (0.84–2.33)	
Used alcohol before or during sex with a male partner in past year	1.72 (0.97–3.06)	
Used illicit drugs before or during sex with a male partner in past year	2.81^f^ (1.49–5.30)	2.43^b^ (1.19–4.98)
Diagnosed with a sexually transmitted infection (STI) in past year	1.97 (0.90–4.31)	
Not tested for HIV in the past year (reference group: had a recent HIV test)	1.72^b^ (1.05–2.84)	1.85^b^ (1.05–3.24)

^a^Values are provided only for factors that were independently associated with unprotected anal sex in the final multivariate model.

^b^*P* < .05.

^c^*P* < .01.

^d^Rooms that are completely dark, often attached to bars, where men have sex.

^e^Private residences where individual rooms are rented by the hour for sexual activity.

^f^*P* < .001.

### Factors independently associated with unprotected anal sex

In the final multivariate model, a number of factors were independently associated with UAI within the target population. UAI with male partners was associated with unemployment (adjusted *OR [AOR]* = 1.87), attendance at an adult movie theater in the past year (*AOR* = 2.21), use of illicit drugs before or during sex with male partners in the past year (*AOR* = 2.43), and not being tested for HIV in the past year (*AOR* = 1.85) (Table 4).

## Discussion

Fifty percent of MSM in our Tijuana sample reported unprotected anal sex with a male partner in the past year. This finding is consistent with previous research that documents high rates of UAI among MSM in other Latin American countries, including Argentina, Brazil, Peru and Ecuador [12-15]. The high prevalence of UAI among MSM in our Tijuana sample suggests that interventions to promote safer sex in this population are urgently needed in this region. We identified four risk factors associated with UAI: illicit drug use before or during sex, frequenting specific venues to meet sex partners, lack of HIV testing, and unemployment.

MSM who patronized adult movie theaters were twice as likely to report UAI with men in the past year compared to men who did not visit this type of venue. Because our survey did not ask participants about the type or frequency of sexual behaviors practiced within specific venues, we cannot conclude that MSM who reported UAI actually engaged in that behavior in adult movie theaters. The latter can only be identified as a venue type where some high-risk men go to meet sex partners. Future studies should gather data on the frequency and type of sexual risk practices that do occur in this type of venue as well as the on characteristics of MSM who patronize adult movie theaters. Previous research with MSM in the U.S. and Australia has found that each type of venue tends to attract men with certain characteristics. For example, Lyons et al. [16] found that younger men were more likely to report anal sex in “sex on premises” venues compared to older men.

Since movie theaters are public settings, there is a greater likelihood that if men engage in sex there, it may be more difficult for them to practice safer sex or to learn each other’s HIV serostatus. Public settings, especially commercial sex venues, have been associated with multiple sexual contacts and group sex [17]. Also, the extent to which alcohol and illicit drugs are used before or during visits to adult movie theaters should be evaluated; previous studies have reported that the use of poppers is common in commercial sex environments [18]. The association between patronization of adult movie theaters and UAI among high-risk MSM in Tijuana suggests that this type of venue could be a target for HIV prevention efforts. HIV prevention messages (on posters and in pamphlets) and free condoms have been shown to be effective in other risk venues associated with sexual activity (e.g., bars/clubs, bathhouses) [19,20]. The advertisement of safer-sex counseling programs in theater venues could be another effective prevention strategy. Also, structural interventions including the availability of condom machines and changes to lighting and space could help to reduce the occurrence of risky sex encounters [17].

MSM who used illicit drugs before or during sex had over two times the odds of having UAI with a man in the past year. Over the past 20 years, the link between the non-injection use of illicit drugs and UAI has been well documented in studies of both HIV-negative [21,22] and HIV-positive MSM [23,24]. In recent years, specific types of illicit drugs have been identified as increasing the odds of high-risk sex among MSM. Cocaine, methamphetamine, and poppers are the most common substances associated with multiple sex partners and unprotected anal sex [25,26]. Studies of MSM in Latin American countries have reported the use of alcohol, marijuana, and cocaine during sexual encounters [12,27,28]. In a study of MSM in Ciudad Juárez, Mendoza-Perez et al. [29] reported that participants who used illicit drugs or alcohol had the highest probability of having risky sex. In the current sample, the most commonly reported illicit drugs used during sex were marijuana and poppers. Reisen et al. also reported that poppers and marijuana were the drugs most frequently used by a sample of Hispanic MSM in the U.S., possibly because they are readily available and inexpensive [30]. Overall, our finding suggests the need for targeted drug intervention that educates MSM about the dangers associated with transitioning to “harder” drugs, such as methamphetamine and cocaine, that are known to increase sexual arousal and the probability of high-risk sex. The association between illicit drug use and high-risk sex also suggests that opportunities for drug treatment for MSM in Tijuana should be expanded.

Being tested for HIV in the past year had a protective effect in our sample, as it was associated with less UAI. This finding is contrary to studies of HIV testing among MSM in developed countries, where higher rates of risk behavior have been associated with seeking out HIV/STI testing [31-33]. In a study of Hispanic men in South Florida, Fernández et al. found that being MSM, having multiple sex partners, and having had sex with someone who had or was suspected of having a STD were among the risk factors associated with HIV testing in the past 12 months, indicating an association between HIV testing and risk awareness [34]. Our finding suggests that MSM who engage in risky sex in Tijuana may have limited access to HIV testing, lower risk awareness and appraisal, or both. In one study of MSM in Peru, 70.9% of the sample had never been tested for HIV, and the most frequently cited reasons were fear of a positive test result and lack of information regarding where to get tested [12]. The challenge for prevention researchers is to increase access to HIV testing, reduce barriers to testing, and convince high-risk MSM to adopt HIV prevention practices that combine consistent use of condoms for anal sex and regular HIV testing. This might be accomplished through more public health messages directed at MSM that promote HIV testing. Another step in the development of health promotion interventions is to identify factors that serve as motivators or barriers to HIV testing among high-risk MSM. In an Australian study, MSM who did not test for HIV were less likely to identify as gay, had fewer gay friends, and spent less time with gay men compared to men who did test for HIV [35]. HIV testing is important as a method of early detection. Testing can help to curtail the spread of HIV through the promotion of prevention practices. It can also have health benefits for the infected individual through initiation of antiretroviral therapy and promotion of condom use. From another perspective, some MSM in our sample were likely to have had access to HIV testing and prevention messages, and yet engaged in UAI during the past year. As shown in studies conducted in the U.S., high rates of UAI among socially connected MSM may be partially explained by “condom fatigue” and frustration with prevention programs that focus exclusively on condom promotion [36-38]. The development of HIV prevention programs that expand upon condom promotion to include other priorities such as communication skills, stress management, coping skills, and broader physical and psychological health issues are warranted.

The rate of unemployment in our sample of MSM was very high (43%) compared to the rate of 7.6% reported for Tijuana in the third quarter of 2011 [39]. Unemployed MSM were approximately two times more likely to report UAI in the past year compared to their employed counterparts. It is plausible that unemployed men are more likely to engage in commercial sex work, which could involve engaging in UAI. The link between unemployment and UAI could also be explained by insufficient funds to purchase condoms or increases in risky health habits. Indeed, previous research has found an association between unemployment and increased cigarette smoking, illicit drug use, and heavy alcohol consumption [40]. It is also possible that unemployed men in Tijuana seek work in the U.S. and subsequently become vulnerable to risk behaviors (UAI, injection drug use) associated with cross-border activity [41]. In the face of a global recession that continues to affect Mexico, it is critical that unemployed MSM in Tijuana be recognized as a vulnerable subgroup that is in urgent need of HIV prevention and intervention.

This study has several limitations. The convenience sample of MSM, which was recruited at a large-scale community event, should not be considered representative of the general population of MSM in Tijuana. In particular, MSM who do not attend gay-oriented public events were not represented in this survey. Non-participants could have differed from volunteers in terms of socio-demographic, psychosocial, and behavioral characteristics. Indeed, there is research to suggest that MSM in Mexico who identify as “heterosexual” are less likely to access HIV resources and are thus in need of targeted prevention efforts [42]. It is also possible that among the men who did attend the event, those with high-risk behaviors and those reticent about their sexual orientation may have been less likely to participate in the survey, which gathered sensitive personal information. Men who did participate in the survey may have been affected by considerations of social desirability and hence may have underreported the extent of their sexual risk and drug use behaviors. If that be the case, we may have underestimated associations between drug use behaviors and UAI. Also, because of Tijuana’s location along the U.S.–Mexico border, our findings may not be generalizable to MSM in other parts of Mexico that are less influenced by cross-border activities and risk behaviors. The one-year time frame used in this study may also have led to inaccurate or faulty recall. Last, this research used participant self-report to ascertain HIV serostatus. Future studies should use laboratory tests of HIV serostatus to examine the association between HIV serostatus and sexual risk behaviors among MSM.

## Conclusions

In summary, this study indicates that MSM in Tijuana remain a risk group that is vulnerable to HIV infection through high-risk sexual practices, including UAI and illicit drug use in the context of sex. Our data suggest the need to conduct a large-scale survey of HIV risk behaviors and HIV/STI prevalence among MSM in this region, which, due to high volume of movement across the international border, should be conceptualized to include the nearby transborder area as well. Such information will help to inform the development of HIV prevention and intervention programs for this population.

Our data also suggest that interventions to promote HIV testing and condom use among MSM in Tijuana should consider venue-specific approaches, as well as focus on drug use in the context of unsafe sex.

## Competing interests

The authors declare that they have no competing interests.

## Authors’ contributions

SBL designed the study and created the survey instrument. RM and AVO participated in the design of the study and helped to write the manuscript. SJS, SAS, and TLP contributed to statistical analysis and data interpretation and helped to write the manuscript. All authors read and approved the final manuscript.

## Pre-publication history

The pre-publication history for this paper can be accessed here:

http://www.biomedcentral.com/1471-2458/12/433/prepub
